# P06 Parents’ perspectives on childhood antibiotic treatment in Ireland—a qualitative study

**DOI:** 10.1093/jacamr/dlag102.012

**Published:** 2026-06-26

**Authors:** Anthony Maher, Eimear C Morrissey, Andrew W Murphy, Gerard J Molloy

**Affiliations:** School of Psychology, University of Galway, Galway, County Galway, Ireland; Centre for Health Research Methodology, School of Nursing and Midwifery, University of Galway, Galway, County Galway, Ireland; Institute for Clinical Trials, College of Medicine, Nursing and Health Sciences, University of Galway, Galway, County Galway, Ireland; Department of General Practice, College of Medicine, Nursing and Health Sciences, University of Galway, Galway, County Galway, Ireland; HRB Primary Care Clinical Trials Network, University of Galway, Galway, County Galway, Ireland; School of Psychology, University of Galway, Galway, County Galway, Ireland

## Abstract

**Background:**

Paediatric respiratory tract infections (RTIs) remain a leading cause of antibiotic use in primary care, despite evidence that most presentations are self-limiting and do not require antimicrobial treatment. While antimicrobial stewardship initiatives have predominantly focused on modifying prescriber behaviour, growing evidence suggests that parental beliefs, expectations, and treatment-seeking behaviours play a critical role in shaping consultation patterns and antibiotic use for childhood illness. In primary care systems characterized by variable access, cost structures, and professional roles, these behavioural drivers are likely to interact with systemic factors in complex ways. The Capability, Opportunity, Motivation–Behaviour (COM-B) model offers a theoretically grounded framework for examining how individual knowledge, environmental constraints, and emotional responses collectively influence antibiotic-related decision-making.

**Objectives:**

To explore parental experiences and perceptions of antibiotic decision-making for childhood RTIs in Ireland, using the COM-B model to identify behavioural drivers and potential intervention targets relevant to antimicrobial stewardship.

**Methods:**

Semi-structured interviews were conducted with 20 parents of children under eight years of age residing in Ireland. Participants were purposively sampled to capture variation in healthcare access and prior antibiotic experience. Interview guides were informed by the COM-B framework and explored parents’ understanding of antibiotic use and antimicrobial resistance (AMR), experiences navigating primary care services, and emotional responses to childhood illness. Interviews were audio-recorded, transcribed verbatim, and analysed inductively using reflexive thematic analysis. Following theme development, findings were mapped to COM-B domains to support theoretical interpretation and identify behavioural mechanisms underpinning treatment-seeking behaviour.

**Results:**

Three interrelated themes were identified, each aligning with distinct COM-B components. First, perceived knowledge gaps in antimicrobial resistance and antibiotic use reflected limitations in psychological capability, with parents expressing uncertainty about when antibiotics were clinically appropriate. AMR was commonly understood as a population-level or future policy issue, rather than an immediate personal concern, particularly in the context of an acutely unwell child. Second, navigating professional gatekeepers captured aspects of physical and social opportunity, highlighting how access to general practitioners, out-of-hours services, pharmacists, and administrative staff shaped both pathways to care and expectations of treatment. Parents described a paradox whereby out-of-hours consultations reduced access barriers while simultaneously increasing the perceived legitimacy of antibiotic prescribing. Pharmacists were frequently viewed as trusted sources of advice, though with limited formal authority. Third, deciding when to act reflected reflective and automatic motivation, as parents balanced a desire for reassurance and professional validation against anxiety surrounding illness escalation, particularly when symptoms persisted or disrupted daily routines. Across themes, timely reassurance and clear communication emerged as central influences on antibiotic-related decisions.

**Conclusions:**

Parental antibiotic decision-making for childhood RTIs is shaped by a complex interaction of capability, opportunity, and motivation operating within the structural realities of primary care. These findings highlight the importance of incorporating behavioural theory into antimicrobial stewardship efforts and underscore the need for socio-culturally informed interventions that address parental concerns alongside systemic access and communication barriers. Recognizing and strengthening the roles of the wider primary healthcare team may support more effective, patient-centred approaches to reducing unnecessary antibiotic use.

